# Pseudogenes and Liquid Phase Separation in Epigenetic Expression

**DOI:** 10.3389/fonc.2022.912282

**Published:** 2022-07-08

**Authors:** Bernard Nsengimana, Faiz Ali Khan, Usman Ayub Awan, Dandan Wang, Na Fang, Wenqiang Wei, Weijuan Zhang, Shaoping Ji

**Affiliations:** ^1^ Laboratory of Cell Signal Transduction, Department of Biochemistry and Molecular Biology, School of Basic Medical Sciences, Henan University, Kaifeng, China; ^2^ School of Life Sciences, Henan University, Kaifeng, China; ^3^ Department of Basic Sciences Research, Shaukat Khanum Memorial Cancer Hospital and Research Centre (SKMCH&RC), Lahore, Pakistan; ^4^ Department of Medical Laboratory Technology, The University of Haripur, Haripur, Pakistan

**Keywords:** liquid phase separation, pseudogenes, RNA modification, epigenetic, cancer

## Abstract

Pseudogenes have been considered as non-functional genes. However, peptides and long non-coding RNAs produced by pseudogenes are expressed in different tumors. Moreover, the dysregulation of pseudogenes is associated with cancer, and their expressions are higher in tumors compared to normal tissues. Recent studies show that pseudogenes can influence the liquid phase condensates formation. Liquid phase separation involves regulating different epigenetic stages, including transcription, chromatin organization, 3D DNA structure, splicing, and post-transcription modifications like m^6^A. Several membrane-less organelles, formed through the liquid phase separate, are also involved in the epigenetic regulation, and their defects are associated with cancer development. However, the association between pseudogenes and liquid phase separation remains unrevealed. The current study sought to investigate the relationship between pseudogenes and liquid phase separation in cancer development, as well as their therapeutic implications.

## Introduction

Cancer remains a global health threat, and its impact on human being has been intense. Excluding melanoma skin cancer, there will be an estimated 19.3 million cancer diagnoses and around 10 million deaths worldwide in 2020 (1). The intricacy of cancer growth pathways, such as changes in the cellular microenvironment, environmental variables, and aberrant gene/epigenetic expressions, influences cancer management challenges. The epigenetic expression has been linked to the development of liquid-liquid phase separation (LLPS). LLPS mediates the epigenetic expression by providing the adaptability of the cellular microenvironment according to the cellular stress (2). LLPS is formed by the interaction between RNAs-RNAs, RNAs-protein, ribonucleoprotein, proteins-proteins, and non-coding RNAs interaction, which subsequently results in the formation of dynamic condensates (3–5).

Pseudogenes have been regarded as defective duplicates of coding genes that lack a functioning gene product for decades (junk DNA). However, using advanced technologies such as RNA sequencing and proteomics analysis, the findings indicate that pseudogenes can influence gene expression by competing with miRNA for the parental gene targets, being translated into peptides, and being transcribed into long non-coding RNAs that participate in other cellular functions (6, 7). The recent tremendous breakthrough in research on cancer has explored the link between pseudogenes and cancer progression. For instance, using cancer proteomics datasets, 1970 novel peptides of pseudogenes were found in tumor tissues, where some pseudogenes-encoded peptides are tumor-specific as pseudogene *RHOXF1P3* is upregulated up to 16 folds in breast cancer (8). Moreover, peptide-encoded non-coding RNAs are linked to cancer formation, with 2044 unique peptides detected in tumor samples and 426 novel peptides found in healthy tissues (8). Along the same lines, *Cx43* pseudogene (*ΨCx43*), a pseudogene of connexin43 (*Cx43*), which is a gap junction protein, is highly expressed in several cancer cell lines but not in normal cell lines (9). **Table 1** summarises the correlation between dysregulated pseudogenes and cancer cell proliferation, migration, and poor prognosis.

**Table 1:** Functions of pseudogenes in different tumors.

Pseudogene	Function	Reference
Pseudogene *PTTG3P*	Its high indicates a poor prognosis breast cancer	(10)
Pseudogene*HMGA1P6* and *HMGA1P7*	Their high expressions promote cancer migration and proliferation	(11)
Pseudogene *CTNNAP1*	Its lower expression promotes cancer by downregulating its cognate gene CTNNAP1 gene expression	(12)
Pseudogene from lncRNA DUXAP10	Its lower expression suppresses the proliferation, migration of pancreatic cancer	(13)
*HMGA1* pseudogenes	*HMGA1* pseudogene enhances the proliferation and migration of the mouse pituitary tumor cell lines	(14)
*DUXAP8* pseudogene	*DUXAP8* pseudogene promotes lung cancers by targeting *EGR1* and *RHOB*	(15)
*DUXAP10* pseudogene	*DUXAP10* pseudogene can serve as a diagnostic, prognostic marker. It promotes hepatocellular carcinoma by activating AKT	(16)
*DUXAP10* pseudogene	*DUXAP10* pseudogene promotes lung cancer by binding with *LSD1* and repressing *LATS2* and *RRAD*	(17)
Pseudogene derived from lncRNA *SFTA1P*	It suppresses the proliferation and migration of gastric cancer	(18)
*DUXAP8* pseudogene	*DUXAP8* pseudogene promotes colorectal cancer proliferation, migration by interacting with *EZH2* and *H3K27me3*	(19)
Pseudogene derived from lncRNA *DUXAP8*	It enhances gastric cancers proliferation and migration by silencing *PLEKHO1*	(20)
*HMGA1P6* pseudogene	*HMGA1P6* pseudogene promotes ovarian cancer by enhancing the expression of *HMGA1*/*2*	(21)
*DUXAP8* derived from lncRNA	It promotes pancreatic carcinoma by silencing *CDKN1A* and *KLF2*	(22)
*DUXAP8* pseudogene	It promotes hepatocellular carcinoma proliferation and migration by sponging *MIR*-*490*-*5P* to enhance the *BUB1* expression	(23)

Apart from their roles in cancer development, pseudogenes affect the formation of liquid phase separation, and various findings show that LLPS affects different epigenetic expression levels. As a result, the abnormal expression of pseudogenes and the formation of LLPS plays a significant role in cancer development. The current study sought to sift through the existing literature to assess the relationship between pseudogenes and LLPS and cancer formation, which may provide a new perspective on approaching cancer development in the future.

## LLPS and Epigenetic Expression Stages

Current research focuses on epigenetic expression as a result of the dysregulation of several variables that contribute to cancer formation. Among these factors, LLPS influences numerous cellular events and subsequently affects different epigenetic expression stages, including chromatin organization, histone modification, transcription factors activation, RNA splicing, non-coding RNAs metabolism, and m^6^A modification.

## LLPS and Chromatin Organization

Genomic DNA is wrapped around histone proteins, forming the more compact and dense complex known as chromatin. This organization regulates various nuclear processes and controls histone modifications or other chromatin-binding proteins (24). The existing literature shows that the nucleosomal array mediates the LLPS, where histone1 enhances the phase separation and leads to the aggregates’ formation, while histone acetylation suppresses the condensate formation (25).

Mechanically, *MeCP2* competes with linker histone H1 and compacts the nucleosomal array to maintain the chromatin structure. A recent study shows that *MeCP2* promotes chromatin condensates by inducing the DNA methylation and LLPS on nucleosomal arrays (26). In the same vein, heterochromatin-binding protein HP1 serves as a transcriptional repressor by binding to methylated lysine 9 residue of histone H3 and assists the chromatin cohesion, promoting aggregation formation in the nuclear state (27). Additionally, numerous membrane-free organelles generated by LLPS (such as paraspeckles and splicing speckles) interact with chromatin *via* their long non-coding RNAs (28).

To efficiently package the genome, the chromatin is settled in three dimensional (3D) structure in the nuclear loci. The study shows that the variation in 3D chromatin structure promotes tumorigenesis (29). Furthermore, the evidence points out the impact of LLPS on the 3D structure, where the suppression of liquid phase separate by 1,6-hexanediol compromises the 3D structure organization in living cells (30–33). Given that histone modification and three-dimensional structure are connected with liquid phase separation, and non-coding RNAs in membraneless organelles interact with chromatin structure, LLPS plays a critical role in chromatin organization.

## LLPS and Transcription Factors

Transcription factors are proteins that can bind to DNA sequences to regulate the rate of mRNA transcription process (34). To achieve transcription precision, the transcriptional factors activate LLPS to concentrate the super-enhancers, enrich the transcriptional factors, and bind the RNA polymerase II. For instance, a transcriptional co-activator, Yes-associated protein (YAP) mediates phase separate condensates formation in the nucleus. Yu et al., 2021 reported that the interferon-gamma induces cancer drug resistance by promoting the nuclear translocation and phase different condensate of YAP, and the disruption of YAP condensates suppresses the tumor growth and promote immune responses (35). Along the same lines, the RNA polymerase II is recruited in phase separation condensates during the initiation of the transcription process; then, the formed aggregate assists the *CycT1* to phosphorylate the carboxy-terminal domain of RNA polymerase II, which subsequently promotes the transcription elongation of polymerase II. Co-activator MED1 and bromodomain-containing proteins like *BRD4* promotes the phase separation at the super-enhancers site (which is dominated by Nanog, Sox, and Oct4) to augment the transcriptional efficacy (36).

The study supports the function of transcription factors in LLPS, demonstrating that synthetic transcription factor aggregates upregulate gene expression up to five fold in various mammalian cell lines and an *in vivo* model (37). TAZ, a transcriptional co-activator with PDZ binding motif, is increased in more than 20% of breast cancers and promotes the growth and spread of cancer cells. TAZ coordinates transcriptional responses by condensing its DNA-binding cofactors and co-activators *via* the LLPS. The deletion of the TAZ coiled-coil domain hinders the formation of LLPS and the ability of the LLPS to begin the expression of its specific genes of interest (38). Based on these findings, LLPS plays a significant role in assuring transcription accuracy by aggregating the various transcription factors.

## Splicing and LLPS

Alternative RNA splicing is the main step in gene expression regulation that allows the production of different messenger RNAs of varied functions from the same gene. Aberrant alternative RNA splicing involves in pathophysiology leading to various diseases, including cardiovascular diseases, immunopathological diseases, neurological diseases, and cancer (39).

The mRNA splicing mediates cellular developmental processes by regulating the liquid phase separation formation. For instance, Embryo defective 1579, which regulates the gene transcription and mRNA splicing, induces the formation of the condensate *in vitro* and *in vivo*, and its suppression affects the global gene expression and mRNA splicing as well (40). Similarly, Ubiquitin Specific Peptidase (*USP42*), which involves the deubiquitination process, uses its C-terminal disordered domain to drive the phase separation of spliceosome components in regulating the various mRNA splicing events. *USP42* integrates the spliceosome component *PLRG1* into nuclear speckles, and its inhibition affects the splicing process, which results in cancer development (41). To the same extent, alternative splicing drives the phase separation of heterogeneous ribonucleoprotein D-like (*hnRNPDL*), which is known to act as a transcriptional regulator. The study reveals that a mutation of the C-terminal disordered domain of *hnRNPDL* promotes the formation of the aggregates and affects the splicing products (42). Kawachi et al. show that splicing of the large exons is associated with phase separation of transcription factors, where the depletion of splicing factors, such as *hnRNP K* and *SRSF3* disrupt the condensate assemblies (43). Le et al. demonstrate that a nuclear protein known as A Kina Anchoring Protein 95 (*AKAP95*), mediates different cellular events, including histone modification, cell-signaling pathways, and RNA splicing can induce phase liquid separate-like aggregates. The *AKAP95* requires the LLPS to control the transcription and RNA splicing effectively, and its defect in the biophysical property and aggregates formation is associated with cancer development (44). Thus, LLPS is an essential regulator of the splicing process, and its aberrant production causes malignancy.

## Role of m^6^A in LLPS Process

m^6^A is the most prevalent mRNA modification, accounting for 25% of all mRNAs. m^6^A affects the mRNA’s placement, translation, and degradation, depending on the included transcript. To achieve these mRNA metabolism processes accurately, m^6^A is assisted by its readers. The m^6^A readers such as *YTHDF1*, *YTHDF2*, and *YTHDF3* can undergo LLPS, and this phase separation process depends on the abundance of m^6^A (45). For instance, *YTHDF2* can recruit the m^6^A -containing transcript into P-bodies for being degraded (46). Moreover, Translocated in LipoSarcoma/Fused, a nuclear RNA-binding that forms the membrane-less aggregates, is affected by various m^6^A modifications in mediating the liquid phase separate condensates (47). Lee et al. show that the m^6^A modification regulates gene expression through liquid phase separate intervention. Furthermore, they reveal that m^6^A on enhancer RNAs display the highly active enhancers and recruit m^6^A reader (*YTHDC1*) to liquid phase separate into aggregates formation, which co-mixes with BRD4. This phase-separated condensate of m^6^A-enhancer RNA and *YTHDC1* reveals the importance of enhancer RNA modification in the LLPS and gene expression control processes (48).

**Figure 1:**
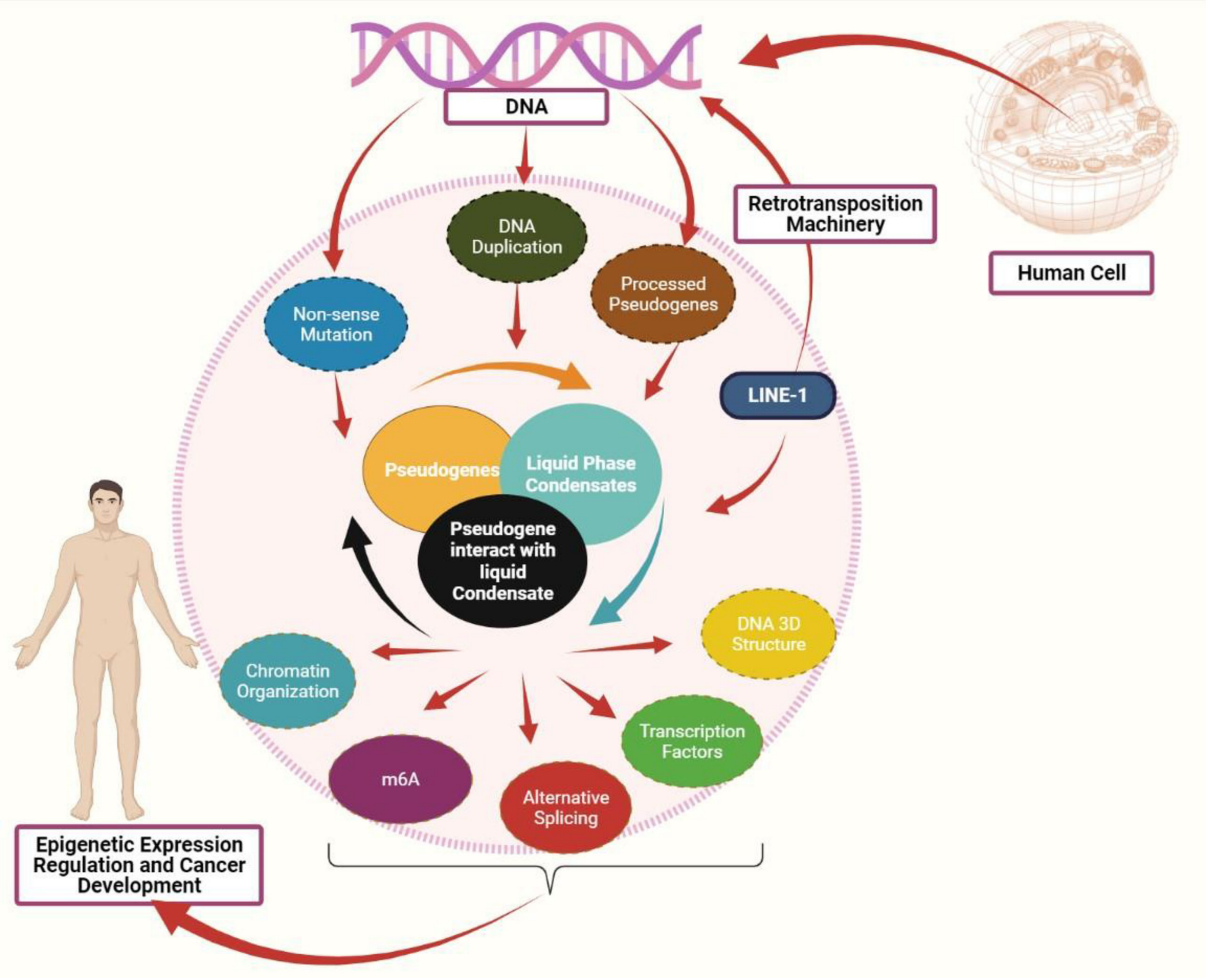
Interaction between pseudogenes and LLPS to mediate cancer: Pseudogenes can be produced in three processes: pseudogenes due to nonsense mutation, pseudogene due to DNA duplication, and processed pseudogenes as the result of retrotransposition machinery. Both LINE-1 and pseudogenes can mediate the liquid phase condensates formation. A defect in pseudogenes expression may affect the liquid phase separate process, eventually affecting the epigenetic mediators, including chromatin organization, DNA 3D structure, transcription factors, alternative splicing, and m^6^A modification. Abnormal expression in these epigenetic mediators promotes cancer development.

Apart from its role in mediating LLPS, the dysregulation of LLPS due to the defect in m^6^A expression is associated with cancer. The m^6^A is required for *YTHDC1* to undergo LLPS and form nuclear aggregates, where the number of nuclear *YTHDC1*-m^6^A aggregates is higher in acute myeloid leukemia than in normal hematopoietic stem cells (49). Based on the aforementioned evidence, m^6^A modification and other epigenetic mediators like chromatin organization and splicing may collaborate to influence the RNA transcript regulation/destiny and mediate the liquid-liquid phase separation process. Their dysregulation contributes to cancer development (**Figure 1**). As the current study fouses on m^6^A, future studies could explore the impact of other RNA modifications to LLPS formation.

## Pseudogenes Formation

Pseudogenes have been considered as non-functional relatives of genes that have lost their protein-coding capability as they lack the necessary sequence required for transcription or translation. More than 10% of the human genome is characterized as pseudogenes and more than 2,075 human genes are denoted by at least one pseudogene. Pseudogenes are frequently generated by DNA duplication, nonsense mutation, or mRNA transcripts that undergo reverse transcription which leads to processed pseudogenes. More than 8,000 processed pseudogenes are associated with Long Interspersed Element-1 (LINE-1 or L1) retrotransposition machinery (50). LINE-1 mRNA achieves its task by using its peptides, namely ORF1p (a nucleic acid chaperone) and ORF2p (an endonuclease and reverse transcriptase). L1 retro-transposition uses its enzymes to reverse the target mRNA transcript and integrate it into the host genome, resulting in a processed pseudogene as a final product (50).

## Role of Pseudogenes in LLPS

LLPS is referred as physical changes of a substance from one state into another, where the homogeneous substance de-mixes into two liquid phase states depending on the threshold of concentration. The factors which influence the threshold concentration of substance include pH, chaperons, ATP, temperature, and posttranscriptional/posttranslational modification (51, 52). Membrane-less organelles are formed by LLPS, once diverse macromolecules such as peptides, coding RNAs, and non-coding RNA connect due to the presence of various interactions such as ionic bonds, hydrogen bonds, and van der Waals forces (53).

The main question is whether non-coding RNAs like pseudogenes can mediate the liquid phase condensates formation. To obtain the answer based on the published literature, the authors first analyzed L1 retrotransposons’ impact on liquid phase separation. As L1 retrotransposons play a critical role in processed pseudogene formation, the crosstalk of L1 retrotransposons to LLPS can indirectly affect the processed pseudogenes formation. Several findings unveil the role of L1 retrotransposons in liquid phase separate formation. The recent study discloses that LINE-1 ORF1 proteins are among other RNA-binding proteins co-localized in stress granules, and LINE-1 ORF1 proteins collaborate with stress granules and processing bodies (P-bodies) to mediate the processed pseudogenes formation (54). L1 is also involved in developing P-bodies since ORF1p co-localizes with non-L1 mRNA that is elevated in P-body granules (55).

Recent study has revealed that LLPS is regulated by intrinsically disordered proteins (IDPs). These misfolded proteins lack a proper 3D structure, which help them to form membrane-less organelles (56, 57). The alpha-synuclein and beta-amyloid are common misfolded proteins in liquid phase aggregate formation (57). As science evolves, a recent study shows that pseudogenes influence the LLPS by mediating the aggregation of these misfolded proteins. For instance, pseudogene *T04B2* regulates the accumulation of beta-amyloid and alpha-synuclein proteins leading to the formation of the aggregates, where the downregulation of pseudogene *TO4B2* intensifies the beta-amyloid and alpha-synuclein protein condensates formation (58). This suggests that the interplay of pseudogenes and misfolded proteins controls the liquid phase separation process.

Besides, pseudogene *ACTBP2* is associated with β-Amyloid (Aβ) depositionin mediating blood-brain barrier permeability, which reveals the interaction between pseudogene and β-Amyloid in regulating barrier permeability (59). Moreover, mRNA purification in P-bodies reveals that 89% is enriched with protein-coding RNAs compared to 67% of non-coding RNAs, with the percentage of pseudogene RNAs among non-coding RNAs co-localized in the P-bodies (60). Similarly, the transcriptomes of lysate granules and stress granules show the presence of various classes of non-coding RNAs, such as long non-coding RNA and pseudogene RNAs as well (61). Another recent study shows a high 7SK small nuclear pseudogene (RN7SKP9) enrichment in stress granules (62). In addition, the nucleolusformed by LLPS involves ribosome biogenesis, cell cycle, DNA damage, and sensing the stress response (63). By using fluorescence-activated cell sorting to isolate the nucleolus and deep sequencing to characterize the nucleolus-associated domains (NADs), pseudogenes are the most enriched in NADs among non-ribosomal RNAs gene, which reveals the impact of pseudogenes in influencing the 3D chromatin structure (64).

Moreover, the dysregulation of the liquid phase separately affects nucleolus formation and leads to different diseases like ribosomopathies, neurodegenerative disease, aging, and cancer (65). The aberrant expression of pseudogenes in NADs can lead to various illnesses, based on evidence that NADs include pseudogenes and the fact that liquid phase condensate impacts the nucleolus to mediate physiology and pathology.

Another membrane-less organelle that formed *via* liquid phase separation is the Cajal body with coilin protein as scaffold protein. Cajal bodies are involved in the cell cycle, cell proliferation, ribonucleoprotein, and telomerase production (66). A recent study shows that the pseudogenes of coilin, coilp1 is accumulated in the nucleus, with strong accumulation in the nucleolus. The same research shows that the protein produced by pseudogene coilp1 has the ability to bind to small Cajal body-specific RNA 2 (*scaRNA2*) and small Cajal body-specific RNA 9 (*scaRNA9*) (67). The scaRNA2 is highly overexpressed in colorectal cancer than normal tissues. The findings reveal that the overexpression of *scaRNA2* competes with miR-342-3p by ending up with the high expression of epidermal growth factor receptor, leading to colorectal cancer chemo-resistance (68). Increased expression of *scaRNA2* also promotes cell proliferation, migration, and invasion in cutaneous squamous cell carcinoma (69).

To summarise, the data indicates that pseudogenes may play a significant role in LLPS by engaging in various misfolded proteins and co-localizing with various membrane organelles such as the nucleolus, Cajal bodies, stress granules, and P-bodies.

## Pseudogenes and LLPS in Cancer

According to the aforementioned findings, LLPS significantly impacts several epigenetic stages, which therefore may mediates cancer development. The dysregulation of different membrane-less organelles formed *via* LLPS, including P-body, Cajal body, and stress granules, promotes cancer progression and metastasis. For instance, the mammary epithelial cells treated with transforming growth factor-beta promote the P-body formation and EMT, while the inhibition of P-body formation suppresses the EMT (70). Bearss et al. show that the enhancer of mRNA decapping 3 (*EDC3*) regulates the cancer cells proliferation and invasion by upregulating the P-body maturation. The inhibition of Pim1 and 3 protein kinases (which phosphorylate the *EDC3*) obstructs the localization of *EDC3* in the P-body (71). Besides, Beneventi et al. show the role of Cajal bodies in cancer progression, where small Cajal body-specific RNAs 15 (*SCARNA15*) regulate the alternative splicing by modulating the pseudouridylation of U2 spliceosomal RNA, which influences the suppressor tumor genes like *p53* and *ATRX*. Suppression of *SCARNA15* downregulates the *p53* expression, followed by cancer cells proliferation (72). Moreover, Adjibade et al. show how the stress granules formation affects cancer drug resistance, where Lapatinib, a tyrosine kinase inhibitor in breast cancer treatment, induces stress granules formation and suppresses the translation initiation by targeting the translation initiation factor *elF2α* (73). These results reveals therefore the importance of liquid phase separation and related membraneless organelles in driving cancer growth.

**Figure 2:**
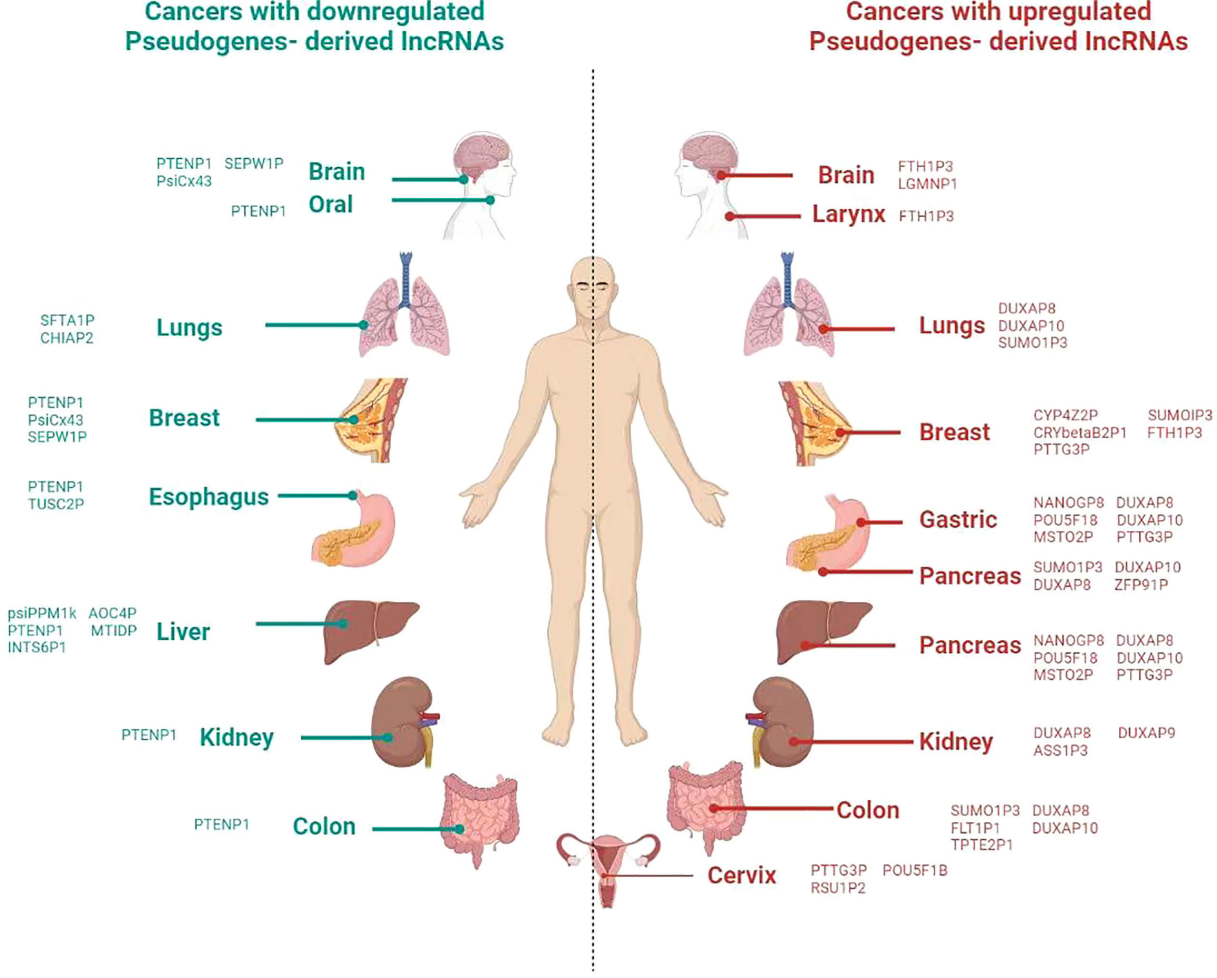
Overview of involvement of pseudogene in different cancer in the human body. Icons of body parts for the refrence are taken from different websources.

Apart from the involvement of LLPS in the epigenetic stages, the recent studies also show the impact of pseudogenes in these cellular processes, including splicing, transcription factors regulation, and chromatin organization. For instance, the splicing of pseudogene *CYP3AP1* to *CYP3A7* induces the formation of *CYP3A7.1L*, which has different functional properties and distribute in tissue specifically than the parental *CYP3A7* enzyme (74). In the line of transcriptional activities, a transcriptional factor, *Foxo3*, is regulated by the expression of *Foxo3* pseudogene (75). Another interesting finding is that the effects of pseudogene on the structure of chromatin are implicated by computational and experimental evidence showing a connection between the modifications of DNA and histones, as well as chromatin remodeling (76). Similarly, *mOct4P4* lncRNA interacts *SUV39H1* (histone methyltransferase) and RNA binding protein *FUS* to target its parental *Oct4* promoter heterochromatin formation (77). Moreover, the recent study shows that the methylation of parental genes and pseudogenes are different in a tissue-specific manner (78). These findings reveal the role of pseudogenes in different cellular processes (**Figure 2**).

Based on the formation of pseudogenes, LINE-1 retrotransposons contribute a lot to produce the processed pseudogenes. Therefore, it is paramount to glance at the impact of these retrotransposons in cancer development before reaching the pseudogenes themselves. The literature shows that about half of all somatic cancers are associated with the integration of retrotransposons, and also LINE-1 retrotransposons compose about 17% of the entire DNA content (78). These evidence brought light to the hypothesis that overexpression LINE-1 retrotransposon might be considered a hallmark of many cancers. Mechanically, abnormal L1 integrations induces the loss of tumor suppressor genes or the amplification of oncogenes *via* breakage–fusion–bridge cycles. Pan-Cancer Analysis of Whole Genomes (PCAWG) project identified 19,166 somatically acquired retrotransposition events, which affected 35% of samples and LINE-1 variation is the most frequent in esophageal adenocarcinoma and the second in colorectal cancers (79). LINE-1 RNA is composed of two non-overlapping open reading frames, encoding two proteins, ORF1p and ORF2p. The expression level of ORF1p is 1000-10,000 times higher than ORF2p, which makes ORF2p less detectable in many cancers. The quantitative analysis shows that the LINE-1 ORF1p encoded peptides are highly overexpressed in uterine corpus endometrial carcinoma, ovarian cancer, and colon cancer compared to their respective normal samples (80).

Moreover, Cancer Genome Atlas (TCGA) analysis shows that expression levels of ORF1p bound mRNAs correlate with the expression of LINE-1 RNA in prostate cancer. In the same study, the findings show that ORF1p interacts with various non-LINE-1 mRNA targets, and these interactions were especially enriched to P-bodies in prostate cancer (55). According to the investigation of the cancer genome atlas and bioinformatics tools, the expression of ORF1p identified in the P-body is connected with the production of pseudogenes and the development of tumors (55). As the LINE-1 retrotransposition involves processed pseudogene formation, the recent study validates that retrotransposition promotes cancer growth. Retrotransposition is related to processed pseudogene insertion in small lung and colorectal cancers, where the pseudogenes are integrated into the promotor or first exon of the suppressor gene (81). Similarly, the retrocopy process is linked to structural variation in human genomes and that several prominent retrocopy insertions are present in malignancies, whereas it is absent in healthy persons (82).

Beta-amyloid fibrils and alpha-synuclein misfolded proteins interact with the pseudogene TO4B2. LLPS is known to be exacerbated by these misfolded proteins. Recent studies reveal the role of these misfolded proteins in cancer development. For instance, the accumulation of amyloid is considered as a part of the tumor microenvironment in glioma, and authors suggest that these accumulations might serve as diagnostic and therapeutic markers (83). The beta-amyloid also promotes the growth and migration of cancer cells by upregulating glial-specific fibrillary acidic protein (GFAP) expression and enhancing angiogenesis. The study also found that amyloid-beta is collected in breast cancer and that its high accumulation is related to high-grade breast cancer (84). The accumulation of amyloid-beta isassociated with the downregulation of tumor suppressor *p53* in cancer (85).

To sum up, retrotransposition elements have a potential role in regulating LLPS and processed pseudogenes. Besides, the dysregulation of pseudogenes expression may affect the misfolded protein regulation. The abnormal retrotransposition elements may affect the pseudogene production, which affects the misfolded protein regulation, resulting in the disorganized liquid phase separate condensate, which can ultimately enhance tumorigenesis.

The findings from bioinformatics databases reveal that modifications and mutations in OCT4 pseudogenes are connected with low survival rate in cancer patient, indicating its potential for cancer prognosis (86). Besides, eight processed *OCT4* pseudogenes, produced by the *POU5F1* gene, exist in different cancer cell lines (87). Mutations in tumour suppressor genes, notably in the PTEN tumour suppressor gene, are thought to be a hallmark of cancer. Methylation of the PTENP1 pseudogene is significantly more prevalent in endometrial cancer than in normal tissues. Due to the possibility of competing for endogenous mRNAs, pseudogene methylation reduces transcription, resulting in the downregulation of the PTEN gene (88). Besides, some pseudogenes are cancer-specific, like *CXADR*-Ψ expression is upregulated in more than 25% of prostate cancer tissues and has no expression in normal tissues (89). For instance, the embryonic *NANOG* (*NANOG1*) gene, which is known as an essential regulator of pluripotency, its deregulation promotes cancer development. The recent study shows that *NANOGP8* (*NANOG*-pseudogene) involves tumorigenesis, and single NANOG1-CRCs form spherical aggregates, indicating its potential in LLPS (90).

Apart from the role of pseudogenes at the transcription level, m^6^A on pseudogenes induce the gene expression accordingly. For instance, some RNAs of processed pseudogenes have more m^6^A levels than their equivalent protein-coding genes, and this modification promotes the degradation of RNA pseudogenes depending on the microRNAs’ involvement (91). Moreover, this modification assists the pseudogenes to mediate tumor growth, where m^6^A modified pseudogene *HSPA7* regulates the immune responses in glioblastoma. The HSPA7 pseudogene triggers the development of YAP1 and LOX, which is followed by macrophage infiltration and the expression of SSP1 (92).

Taken together, the pseudogenes can influence gene expression and cancer development due to their presence in different epigenetic factors like LLPS, transcription process, m^6^A modification, regulating the misfolded proteins, and some of them are cancer-specific (**Figure 2**).

## Clinical Utility of Pseudogenes and LLPS in Cancer Management

Several challenges have been identified in cancer management, including cancer relapse and chemo-resistance. It is known that the best treatment relies on accurate diagnosis. To overcome cancer therapy failure, new indicators could be investigated. Nevertheless, the new findings demonstrate that pseudogenes are elevated in cancer tissues than their normal counterparts, and their overexpressions are related to poor prognosis. For instance, pseudogene *DUXAP10* is upregulated in different types of cancer (93). Besides, high expression of *ANXA2* pseudogene induces a shorter overall survival in hepatocellular carcinoma patients (94). Similarly, increased expression of *HSPB1P1* pseudogene is associated with poor prognosis in renal cell carcinoma (95). Thus, it reveals that pseudogenes can serve as novel markers in cancer diagnosis.

Additionally, a recent study discovered that LLPS-related genes are overexpressed in various malignancies, including ovarian epithelial carcinoma (96). Moreover, aberrant of LLPS is associated with cancer drug resistance in multiple myeloma. The mechanism behind this chemo-resistance is that the overexpression of histone methyltransferase NSD2 promotes the elevated steroid receptor coactivator-3 (SRC-3) by stimulating its aggregates formation *via* LLPS. Targeting this interaction using an inhibitor, SI-2, enhances the BTZ treatment functionality and overcomes this chemo-resistance (97). Along the same lines, the inhibition of core regulatory circuity, which interacts with super-enhancer to mediate LLPS at the transcriptional level, uses H3K27 demethylase inhibitor, GSK-J4, re-sensitizes the chemotherapy in osteosarcoma (98).

Following that, pseudogene can affect the liquid phase separation process. Abnormal pseudogene expression and LLPS formation can promote cancer growth, resulting in a low survival rate, a poor prognosis, and chemo-resistance (99). Therefore, impact of pseudogenes and LLPS may be used as biomarkers in cancer diagnosis, and targeting LLPS may give a novel therapeutic approach in near future.

## Conclusions and Future Perspectives

Since the last few decades, much effort and research have been made to unravel the interplay of the small biomolecules involved in carcinogenesis. However, the connection between pseudogenes and the liquid phase remains fragmented. The purpose of this review is to examine the relationship between LLPS and pseudogenes and their potential impacts on cancer development. Based on the findings, pseudogenes considered junk DNA, are involved in different gene expression stages, including transcription process, post-transcription modifications, and regulating the liquid phase condensates formation.

The dynamicity of liquid phase separation (its property to mix and de-mix depending on cellular stress) greatly impacts gene epigenetic expression regulation. Its defect may result in different diseases, including infectious diseases, neurodegenerative disorders, and cancer. Since tumors may change morphology and respond in unpredictable ways, it’s critical to think about how pseudogenes (some of which are only found in tumor cells) can affect future cancer detection and treatment. Moreover, it will be interesting to explore the potential biomarkers based on LLPS in different diseases diagnosis and treatment. For instance, studies could investigate whether scaffold protein expression of the membraneless organelles can be used to differentiate cancer cells from normal cells. Furthermore, based on the fact that pseudogenes are considered as long non-coding RNAs, and their impact to LLPS is tremendous, it would be for paramount to assess the impact of microRNAs to the LLPS in the future.

## Author Contributions

BN and FAK write the first draft, refined, edited, and revised the manuscript, designed the tables and figures. UAA, DW, and NF formatted and contributed to the materials organization and final editing of the manuscript. WW, WZ, and SJ conceptualized, designed and supervised the study. All authors listed have made a substantial, direct and intellectual contribution to the work and approved for publication.

## Funding

This work was supported by the National Natural Science Foundation of China (No. 31371386 SP.J).

## Conflict of Interest

The authors declare that the research was conducted in the absence of any commercial or financial relationships that could be construed as a potential conflict of interest.

## Publisher’s Note

All claims expressed in this article are solely those of the authors and do not necessarily represent those of their affiliated organizations, or those of the publisher, the editors and the reviewers. Any product that may be evaluated in this article, or claim that may be made by its manufacturer, is not guaranteed or endorsed by the publisher.

## Abbreviations

3D, Three dimensional; AKAP95, A Kina Anchoring Protein 95; *Cx43*, Connexin43; EDC3, Enhancer of mRNA decapping 3; hnRNPDL, Heterogeneous ribonucleoprotein D-like; IDPs, Intrinsically disordered proteins; LINE-1 or L1, Long Interspersed Element-1; LLPS, Liquid-liquid phase separation; NADs, Nucleolus associated domains; P-bodies, Processing bodies; PCAWG, Pan-Cancer Analysis of Whole Genomes; RN7SKP9, 7SK small nuclear pseudogene; scaRNA2, small Cajal body-specific RNA 2; TAZ, Transcriptional co-activator with PDZ binding motif; USP42, Ubiquitin Specific Peptidase; YAP, Yes-associated protein.
